# Agreement between two photoplethysmography-based wearable devices for monitoring heart rate during different physical activity situations: a new analysis methodology

**DOI:** 10.1038/s41598-022-18356-9

**Published:** 2022-09-14

**Authors:** Carla Alfonso, Miguel A. Garcia-Gonzalez, Eva Parrado, Jessyca Gil-Rojas, Juan Ramos-Castro, Lluis Capdevila

**Affiliations:** 1 Laboratory of Sport Psychology, Department of Basic Psychology, Universitat Autònoma de Barcelona (UAB), 08193 Bellaterra, Barcelona Spain; 2 Sport Research Institute, Universitat Autònoma de Barcelona (UAB), Bellaterra, Spain; 3 Group of Biomedical and Electronic Instrumentation, Department of Electronic Engineering, Universitat Politècnica de Catalunya (UPC), Barcelona, Spain

**Keywords:** Biomarkers, Biotechnology, Biomedical engineering, Public health

## Abstract

Wearables are being increasingly used to monitor heart rate (HR). However, their usefulness for analyzing continuous HR in research or at clinical level is questionable. The aim of this study is to analyze the level of agreement between different wearables in the measurement of HR based on photoplethysmography, according to different body positions and physical activity levels, and compared to a gold-standard ECG. The proposed method measures agreement among several time scales since different wearables obtain HR at different sampling rates. Eighteen university students (10 men, 8 women; 22 ± 2.45 years old) participated in a laboratory study. Participants simultaneously wore an Apple Watch and a Polar Vantage watch. ECG was measured using a BIOPAC system. HR was recorded continuously and simultaneously by the three devices, for consecutive 5-min periods in 4 different situations: lying supine, sitting, standing and walking at 4 km/h on a treadmill. HR estimations were obtained with the maximum precision offered by the software of each device and compared by averaging in several time scales, since the wearables obtained HR at different sampling rates, although results are more detailed for 5 s and 30 s epochs. Bland–Altman (B-A) plots show that there is no noticeable difference between data from the ECG and any of the smartwatches while participants were lying down. In this position, the bias is low when averaging in both 5 s and 30 s. Differently, B-A plots show that there are differences when the situation involves some level of physical activity, especially for shorter epochs. That is, the discrepancy between devices and the ECG was greater when walking on the treadmill and during short time scales. The device showing the biggest discrepancy was the Polar Watch, and the one with the best results was the Apple Watch. We conclude that photoplethysmography-based wearable devices are suitable for monitoring HR averages at regular intervals, especially at rest, but their feasibility is debatable for a continuous analysis of HR for research or clinical purposes, especially when involving some level of physical activity. An important contribution of this work is a new methodology to synchronize and measure the agreement against a gold standard of two or more devices measuring HR at different and not necessarily even paces.

## Introduction

Wearable technology uses smart electronic devices that are worn close to or on the surface of the skin, to detect and analyse body signals and/or ambient data and transmit it to the phone^1^. In the last decade, wearable devices have become more comfortable, lightweight, and cost-effective for assessing health behaviour, and at present, they have shown potential applications in personal recovery, sleep and fitness, as well as medical surveillance, non-invasive medical care, and mobile health-wellness monitoring^2^. With an expected number of connected wearable devices of more than one billion by 2022^3^, the number of companies developing such technology is growing speedily, and the need to test the accuracy of data collected by these devices increases accordingly. The physiological signals recorded by wearables can have immediate clinical, research and practical impact in the monitoring of fitness and medical conditions, so there is a need to determine whether their measurements are within clinical limits of agreement^4^. One of the physiological parameters those wearable devices measure, and whose validity has been tested compared to a gold standard, is heart rate (HR).

HR is a measure of cardiac activity usually expressed as the number of beats per minute (bpm). The current gold-standard method for assessing HR is the standard 12-lead electrocardiogram (ECG), while HR measurements from wrist-worn wearables are predominantly obtained from photoplethysmography (PPG). PPG is an optical measurement technique that allows to collect volumetric changes in blood perfusion under the skin using a light emitter and a photodetector. HR research has been limited by the lack of ecological validity, inability to collect data during a representative time span and the obtrusiveness of the HR measurements. Fortunately, the present irruption of wearables has the potential to increase and improve the research on HR provided that the measurements are valid. In the future, continuous wearable-based technology has great potential for helping users to monitor their health, as well as impacting clinical and research settings, by guiding healthcare decisions and medical interventions. For this reason, it is important to prove the accuracy and suitability of wearables for the assessment of HR. To date, studies exploring the accuracy of wearables’ HR compared to ECG indicate that, on average, wearables slightly underestimate absolute HR^5–7^ with the Apple Watch having slightly greater accuracy than other devices such as Fitbit^4,8^. The intensity of body movement seems to affect the detection of HR, as well as the position where devices are worn, with the wrist being particularly susceptible to movement and to corrupting the PPG signal and affecting the accuracy of the estimation^9^. Overall, it is known what PPG lags behind ECG when it comes to HR detection^10^, yet it is still interesting to keep testing the accuracy of PPG because it is less intrusive, low cost and convenient way to detect cardiac changes than ECG^11^.

The accuracy of HR in situations that involve movement seems to depend on two factors: motion complexity and level of physical activity^4^. Wearables are more accurate during rest, low intensity exercise^7,12^ and locomotor activities characterized by repetitive movements (eg, cycling, walking or running)^7,12–14^. Some research shows that absolute error during activity is higher with resistance training exercises, with inherently more complex movements, being more inaccurate (35% accuracy) compared to aerobic exercise (92% accuracy)^15,16^.

Recent guidelines and recommendations complain that there is a lack of transparency from manufacturers on describing the underlying signal processing and on disclosing the HR data measured by their devices^4,17^. This lack of information complicates the comparison procedure among wearables of different manufacturers. To validate a wearable, comparisons among two or more time series quantifying the HR in a selected time scale must be made. For the sake of comparison, the time series must be properly synchronized and represent the HR at some time scale (i.e. by averaging the sampled data during the same time span). For the sake of automatic synchronization and validity assessment, the majority of validation studies include an interpolation procedure to resample the information of the devices to be compared at the same sampling frequency^17^. Moreover, a large proportion of studies only compare the average of the HR of the overall recording. Hence, they use a time scale of some minutes (often 5 min) losing the opportunity to check if the wearable can correctly track variations of HR along the recording. Interpolation adds fictitious data to the sparser sampled time series (generally, to the time series obtained from the wearables) while measuring at only one time scale narrows the scope of the validation procedure.

The aim of this study is to determine the validity of the measured HR, as a key health and fitness measurement, from two of the most popular wearables: the Apple Watch and Polar Vantage, under different activities. The study will compare these devices to a gold-standard ECG in different positions to account for motion complexity and level of physical activity. Moreover, the study proposes a new methodology for validity testing that can be employed to gain more insight on signal processing differences among devices. The proposed methodology avoids the interpolation of the HR data to compare with the gold-standard measurements and it advocates for the device’s comparison over different time scales to track variations of HR along the recording.

## Materials and methods

For this study, two heart rate photoplethysmography-based wearable measurement devices were compared against the beat-to-beat heart rate obtained by a reference ECG system. The two wearable devices were the Apple Watch S6 (Apple, Cupertino, CA, USA) (AW) and the Polar Vantage M2 (Polar Electro Oy, Kempele, Finland) (PV). The ECG was acquired using a Biopac MP36 data acquisition system (Santa Barbara, CA, USA) using a sampling frequency of 1 kHz and limiting the bandwidth of the amplifier between 0.5 Hz and 150 Hz. The accurate beat-to-beat heart rate obtained with this system was the gold standard measure (GS) for comparison against the other systems. Both AW and PV provide estimates of HR obtained by filtering and processing the detected heartbeats using photoplethysmographic techniques and non-disclosed and proprietary algorithms. The PV provides HR updates each second while the AW provides HR samples at more unstable times, typically ranging from 1 to 9 s.

### Participants

Twenty participants started the study but 2 were rejected due to poor quality of the ECG signal. Hence, eighteen university students (10 males, 8 females), with a mean age of 22 ± 2.45 years, were included in the study. All participants were volunteers and provided written consent. Descriptive statistics of the participants are shown in Table 1. Privacy was assured for all participants as regards all data collected. The study was conducted according to the guidelines of the Declaration of Helsinki and approved by the local Ethics Commission for Human Experimentation of the Autonomous University of Barcelona (protocol code CEEAH-5745).

**Table 1:** Descriptive statistics for participants.

	Mean ± SD
Age (years)	22 ± 2.45
Height (cm)	172.21 ± 8.95
Women	165.5 ± 7.18
Men	176.9 ± 6.97
Weight (kg)	65.0 ± 9.99
Women	57.25 ± 9.21
Men	71.2 ± 5.16

### Procedure

A within-subject design was used in this study. Participants were contacted via email or Twitter. The study was conducted in one session. Before starting the session, participants completed an informed consent form. Weight and height were measured before starting the HR recordings.

The measuring HR devices (GS, AW and PV) were placed on each participant. The ECG electrodes for GS measurement were attached near the clavicula (one at each end), while the reference electrode was placed at the jugular notch, ensuring an ECG signal good enough for proper QRS detection while allowing the movement of the participants from place to place according to the measurement protocol. The AW was placed on the right wrist and the PV on the left wrist. Each participant was asked to remain in a lying position for 5 min (lying activity), then to seat on a chair for 5 min (sitting activity), stand for 5 min (standing activity), and finally walk on a treadmill without inclination at a speed of 4 km/h for another 5 min (walking activity). When moving from one position to another, 30 s were allowed to let the signal stabilize. The researchers manually annotated the starting and ending times of the activities.

The HR series of the PV device were downloaded from the Polar Account webpage after syncing the PV device with the Polar Flow service. The data format was a text file with two columns for each session (timestamp and HR data). For the HR series of the AW, the AW was automatically synced with an iPhone 10. All health data of the iPhone was exported to an .xml file and the HR series were extracted from the file with a short script written in Matlab.

### Signal processing

**Figure 1:**
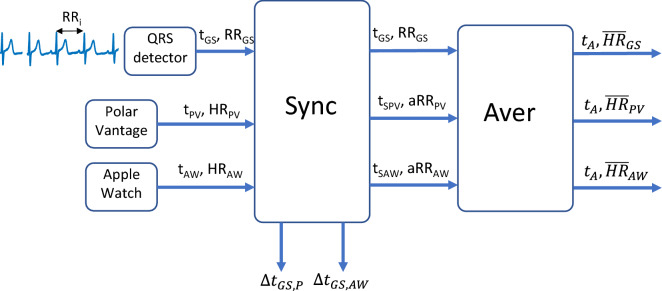
Signal processing stages. The Sync block synchronizes the time stamps of the point heart rate estimates for the alternative measurement methods while the Aver block provides an average of point estimates for each method at the same reference time stamp. See text for further details.

Figure 1 shows the main stages of the signal processing procedure. A QRS detector detects the R peak locations from the ECG of the GS system. These locations are used to generate a timestamp and a gold standard RR time series (t_GS_ and RR_GS_, respectively). On the other hand, the PV and AW systems provide their timestamps (t_PV_ and t_AW_) in correspondence with their point estimates of HR. These estimates are easily converted to average heart periods (aRR_PV_ and aRR_AW_). The first step is to synchronize the timestamps of the studied measurement systems with respect to the gold standard. This procedure estimates the delay between t_GS_ and t_p_ or t_AW_ by minimizing the error between the point estimates and the RR_GS._ Synchronization creates new timestamps for the PV and AW systems (t_SPV_ and t_sAW_). After synhronization, the averaging block identifies the RR_GS_ intervals and aRR_PV_ and aRR_AW_ point estimates that lie inside a certain interval of length *t*_*s*_ (averaging time), compute their mean values and converts them to mean HR estimates. Because these estimates are obtained at the same temporal location (t_A_), these values can be directly compared to measure the agreement with respect to the GS.

The QRS complexes were detected using the same procedure described in^18^ that starts with a first estimation of the QRS locations using a Pan-Tompkins QRS detector^19^. The QRS locations are further refined using a matching pattern technique. After QRS detection, the QRS locations were further refined by looking for outliers and correcting them using the same approach of^20^. See a more detailed description of the QRS detection and outlier detection and correction in Appendix A1 in the supplementary materials. At last, the QRS detector, including the artifact correction, provides the RR_GS_ time series and its corresponding timestamps t_GS_ time series for each volunteer.

AW and PV systems provide their heart rate time series (HR_AW_ and HR_PV_) expressed in beats per minute (BPM) at different interval times (not necessarily regular) as measured by their timestamps (t_AW_ and t_PV_) in milliseconds. Because timestamps for the gold standard are referred to the beginning of the ECG recording, the first manipulation of the t_AW_ and t_PV_ time series was subtracting to every timestamp the initial timestamp corresponding to the first provided HR measurement.

Note that at this stage, the three different timestamp time series are differently sampled and they can include significant delays due to different starting measurement times, clock errors and delays introduced by each measuring system. The synchronization procedure estimates these delays and it is described in detail in Appendix A2 in the supplementary materials. The output of this procedure performs an straightforward transformation of the HR_AW_ and HR_PV_ time series to averaged RR period time series (aRR_PV_ and aRR_AW_) and provides new timestamps (t_SPV_ and t_SAW_) synchronized with t_GS_.

1 $$AWaRR\left(j\right)=\frac{60000 \,ms/minute}{AWHR(j)}$$

After synchronization, time series are split in four parts corresponding to the four different activities (lying, sitting, standing and walking). The partition is made considering the manual annotations and starts at 10 s after the annotation of the beginning of the activity and ends 10 s before the annotation of the end of the activity.

The first approach to assess the agreement compares the mean HR for each activity and devices obtained from the average the RR time series against the HR obtained from the GS for the same individual and activity. Hence, after activity partition, mean HR in beats per minute (BPM) were obtained for each device, activity and subject as

2 $${mHR}_{d}^{s,a}=\frac{60000}{{mRR}_{d}^{s,a}}$$

where ${mRR}_{d}^{s,a}$ is the mean RR time series (in ms) averaged using all the available samples for subject *s* during activity *a* while measuring with device *d* (averaging time series aRR_PV,_ aRR_AW_ or RR_GS_ for PV, AW or GS systems respectively). Each of these averages covers a time interval of around 5 min.

These mean HR values were analyzed with the IBM SPSS Statistics package for Mac OS (version 25), and the significance threshold was set at p < 0.05. First, the Kolmogorov–Smirnov test was applied to prove that all HR values presented normality of the distributions, for all activities. Analysis of variance (ANOVA) for repeated measures was applied to compare the averages of HR values on approximately 5 min periods between the three different devices for each situation. Bonferroni contrast tests for repeated measures was applied to compare the differences between HR mean values and to calculate the 95% confidence interval. The effect size for ANOVA (repeated measures) was also analyzed from the parameter partial eta-squared (η_p_^2^); benchmarks provided by^21^ were used to define small (η^2^ = 0.01), medium (η^2^ = 0.06), and large (η^2^ = 0.14) effects. G*Power (v3.1; Heinrich-Heine-Universität Düsseldorf, Düsseldorf, Germany) was used to analyse statistical power for analysis of variance (ANOVA) for repeated measures. To calculate the statistical power, we considered an *n* = 18, four factors of repeated measurements (lying, sitting, standing and walking), the overall effect size calculated for all factors and the lowest correlation observed between devices for that factor. Pearson correlation analyses were performed to test HR bivariate associations between pairs of devices for each situation.

Because the RR_GS_ time series reflects the beat-to-beat variability of the heart rate while aRR_AW_ and aRR_PV_ have some kind of filtering and averaging, in order to provide a fair agreement measurement, including the tracking of HR changes, the three RR time series were smoothed by averaging their samples using different averaging times (*t*_*s*_). The averaging procedure for each subject, device and activity looks for the timestamps of the three systems (t_GS_, t_SAW_ and t_SPV_) that are included in the interval [*t*, *t* + *t*_*s*_] and outputs the mean value of the RR time series (either for RR_GS,_ aRR_AW_ or aRR_PV_) that correspond to these timestamps. The procedure considers as the starting time (*t*_*o*_) the first timestamp of the t_GS_ that corresponds to the beginning of the activity under study. In this work we have considered averaging times from 5 to 60 s (in steps of 1 s). This procedure provides estimates of the averaged RR time intervals at the same instants for the three systems. Because AW and PV provide their measurements as heart rate in BPM, for each subject (*s*), activity (*a*) and averaging time (*t*_*s*_) the following three time series were obtained:

3 $$\overline{{HR }_{\mathrm{GS}}^{s,a,{t}_{s}}}\left(m\right)=\frac{60000}{\overline{{aRR }_{GS}^{s,a,{t}_{s}}\left(m\right)}}$$

4 $$\overline{{HR }_{\mathrm{AW}}^{s,a,{t}_{s}}}\left(m\right)=\frac{60000}{\overline{{aRR }_{AW}^{s,a,{t}_{s}}\left(m\right)}}$$

5 $$\overline{{HR }_{\mathrm{PV}}^{s,a,{t}_{s}}}\left(m\right)=\frac{60000}{\overline{{aRR }_{PV}^{s,a,{t}_{s}}\left(m\right)}}$$

where $\overline{{aRR }_{GS}^{s,a,{t}_{s}}\left(m\right)}$, $\overline{{aRR }_{AW}^{s,a,{t}_{s}}\left(m\right)}$ and $\overline{{aRR }_{PV}^{s,a,{t}_{s}}\left(m\right)}$ are the mean value of the RR_GS_, aRR_AW_ and aRR_PV_ time series in the interval [*t(m)*, *t(m)* + *t*_*s*_] being *t*(*m*) = *t*_*o*_ + (*m-1)*·Δ*t* for subject *s* and activity *a*. In this work we have chosen Δ*t* as 1 s. If for a certain combination of *t*_*s*_and Δ*t* there is a device that does not have any timestamp inside the interval, the computation in this interval is skipped. The Appendix A3 in the supplementary materials shows and example on how the averaging procedure is made. Note that this methodology can be easily modified to allow for other location statistics such as the median or the mode of the time series by simply computing these statistics in the intervals of length *t*_*s*_ instead of the arithmetic mean. The averaging for longer *t*_*s*_allows to study the agreement of the devices when mean heart rate is the target indicator by smoothing all causes of heart rate variability (HRV). Analysis using shorter *t*_*s*_allows to study how fast the devices can track changes in heart rate. Nevertheless, for short *t*_*s*_the normal heart rate variability of the subject will reduce the agreement between devices.

Now that the three time series are sampled at the same intervals, agreement analysis for different averaging times can be performed. Results are based on quantifying Bland–Altman plots^22,23^ by comparing the samples of either AW or PV with the GS. These plots change with activity, subject and averaging time and are scatterplots where each point corresponds to:

6 $${\left(x\left(m\right),y(m)\right)}_{ts}^{s,a}=\left(\frac{\overline{{HR }_{\mathrm{GS}}^{s,a,{t}_{s}}}\left(m\right)+\overline{{HR }_{\mathrm{DEV}}^{s,a,{t}_{s}}}\left(m\right)}{2},\overline{{HR }_{\mathrm{GS}}^{s,a,{t}_{s}}}\left(m\right)-\overline{{HR }_{\mathrm{DEV}}^{s,a,{t}_{s}}}\left(m\right))\right)$$

and $\overline{{HR }_{\mathrm{DEV}}^{s,a,{t}_{s}}}\left(m\right)$ can be either $\overline{{HR }_{\mathrm{AW}}^{s,a,{t}_{s}}}\left(m\right)$ or $\overline{{HR }_{\mathrm{PV}}^{s,a,{t}_{s}}}\left(m\right)$ depending on the systems intended to be compared. BA will be computed by pooling the data for every subject and for different averaging times. Because the differences between systems ($y(m)$) may be not symmetrically distributed, the percentiles 2.5% and 97.5% of the differences were computed for the pooled Bland–Altman for each activity and averaging time as surrogate measures of the limits of agreement (LoA) of the BA. It is expected that the dispersion of BA, measured as the difference between percentiles, will decrease by increasing the *t*_*s*_because of the progressive smoothing of heart rate variability and random noise.

The median of the differences was employed as a quantifier of the bias between measurements. Statistical significance of the difference between biases when comparing different devices, averaging times or activities were assessed using the non-parametric Wilcoxon Rank Sum Test^24^.

Statistical significance of differences in the spreading of the BA when comparing different devices, averaging times or activities were assessed by comparing the standard deviations of the differences using the non-parametric Ansari-Bradley Test^25^ after removal of the median value of the difference for each BA.

Synchronization, averaging of time series and BA plot analysis and their associated statistical tests were performed with MATLAB® (R2021 Update 3 for 64 bits Windows).

### Institutional review board statement

The study was conducted according to the guidelines of the Declaration of Helsinki, and approved by the local Ethics Commission for Human Experimentation (protocol code CEEAH-5745).

### Informed consent

Informed consent was obtained from all participants involved in the study.

## Results

Table 2 shows the results of analysis of variance (ANOVA) for repeated measures, comparing 5-min mean HR between devices as defined in (1), in the different situations (lying, sitting, standing and walking). Bonferroni contrast tests was applied to compare the differences between pairs of devices regarding HR mean, as well as to calculate the 95% confidence interval when ANOVA shows significance.

**Table 2:** HR mean obtained from GS, AW and PV (mean ± SD) in bpm.

Activity	Biopac (GS)	Apple Watch (AW)	Polar Vantage (PV)	ANOVA (*p)*
Lying	62,92 ± 10,72	62,62 ± 10,59**	61,87 ± 10,68***	< .001
Sitting	71,18 ± 12,39	70,94 ± 12,40	69,95 ± 12,36	.278
Standing	77,10 ± 12,97	76,92 ± 13,10	72,95 ± 14,10*	.019
Walking	86,48 ± 13,37	86,76 ± 13,24	90,91 ± 19,42	.358

*p < .05;

Significance is shown according to Bonferroni contrast tests applied to compare the differences between HR mean values from an ANOVA analysis between wearables.

**p < .01; ***p < .001. Significant difference compared to GS (Bonferroni contrast test for repeated measures). Statistical Power: *π* = 0.99.

**Figure 2:**
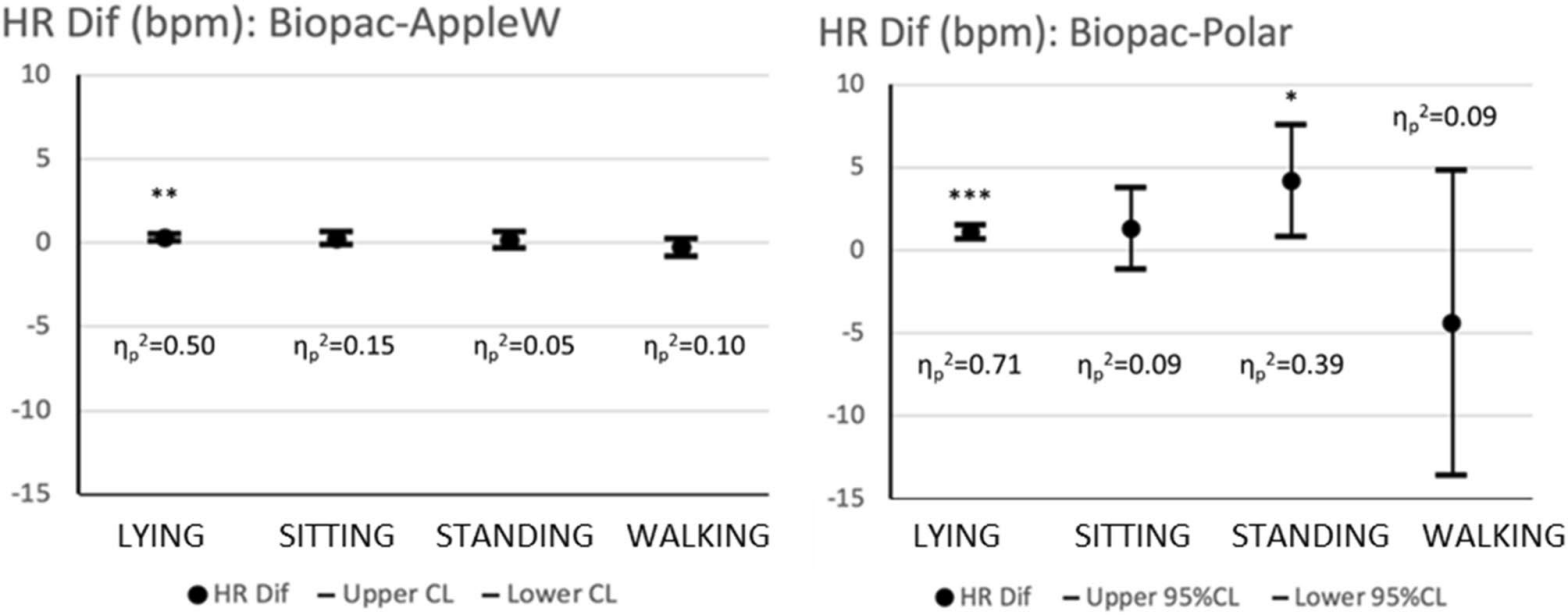
Differences of HR mean values between GS and the other devices. The mean value of the differences and their 95% confidence interval are represented as well as the significance of Bonferroni Contrast Test *p < .05; **p < .01; ***p < .001 and the η_p_^2^ measuring the effect size.

The differences of HR mean values between devices can be calculated from the data in Table 2. Figure 2 represents a summary of the results of Bonferroni contrast tests comparing these differences and calculating the 95% confidence interval. The effect size for ANOVA is also analyzed. The statistical power for this analysis was *π* = 0.99, considering *n* = 18, four factors of repeated measurements (lying, sitting, standing and walking), an overall effect size of 0.37 and the lowest correlation observed between devices of 0.652.

Table 3 shows that AW presents a high correlation of HR mean values with the GS in all situations (always higher than 0.998), while the correlation of PV and GS decreases as the level of physical activity increases.

**Table 3:** Pearson correlation coefficients (r) of HR mean values between Biopac (GS), Apple Watch (AW) and Polar Vantage (PV) systems for the four activities (n = 18).

Activity	Lying		Sitting		Standing		Walking	
Wearable	Apple (AW)	Polar (PW)	Apple (AW)	Polar (PW)	Apple (AW)	Polar (PW)	Apple (AW)	Polar (PW)
Biopac (GS)	1.000**	.998**	.999**	.950**	.998**	.925**	.998**	.652*
Apple (AW)	–	.998**	–	.960**	–	.929**	–	.677*

Significant differences: *p < .01; **p < .001**

**Figure 3:**
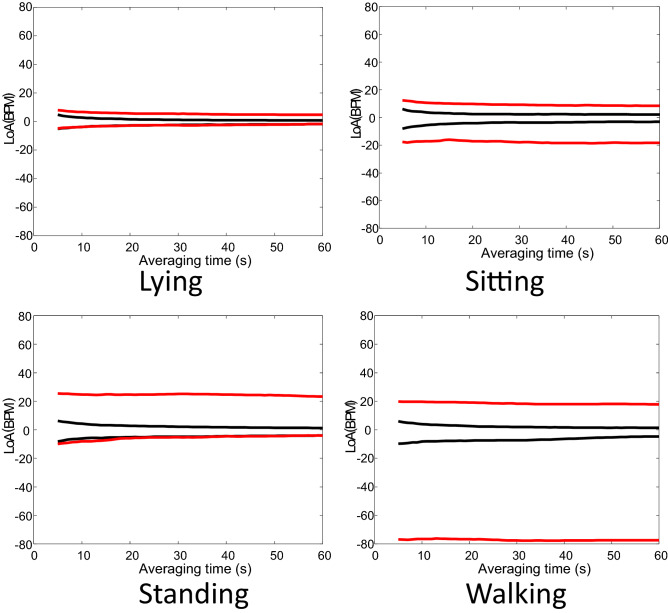
Change in the Limits of agreement (LoA) with 2.5% and 97.5% percentiles of the Bland–Altman plots with respect to the averaging time (from 5 to 60 s) for the four activities. Red and black lines correspond to the PV and AW devices respectively.

In Appendix B1 can be consulted the BA plots defined by (5) and obtained for each activity by averaging in time intervals of length *t*_*s*_ (using a Δ*t* = 1 s to update the limits of the time intervals) and by pooling all the subjects for selected averaging times are shown. Nevertheless, all these results can be summarized in Fig. 3 that shows how the limits of agreement (LoA), defined as the percentiles 2.5% and 97.5% of the differences with respect to the GS, evolve with the averaging epoch length (*t*_*s*_) for the AW (in black) and the PV (in red). As seen in Fig. 3, the AW has generally tighter LoA than the PV for every averaging time. For the AW, as the averaging time increases, the limits of agreement narrow, whereas for the PV, the dependency of the LoA with the averaging time is not so noticeable, especially while walking.

**Figure 4:**
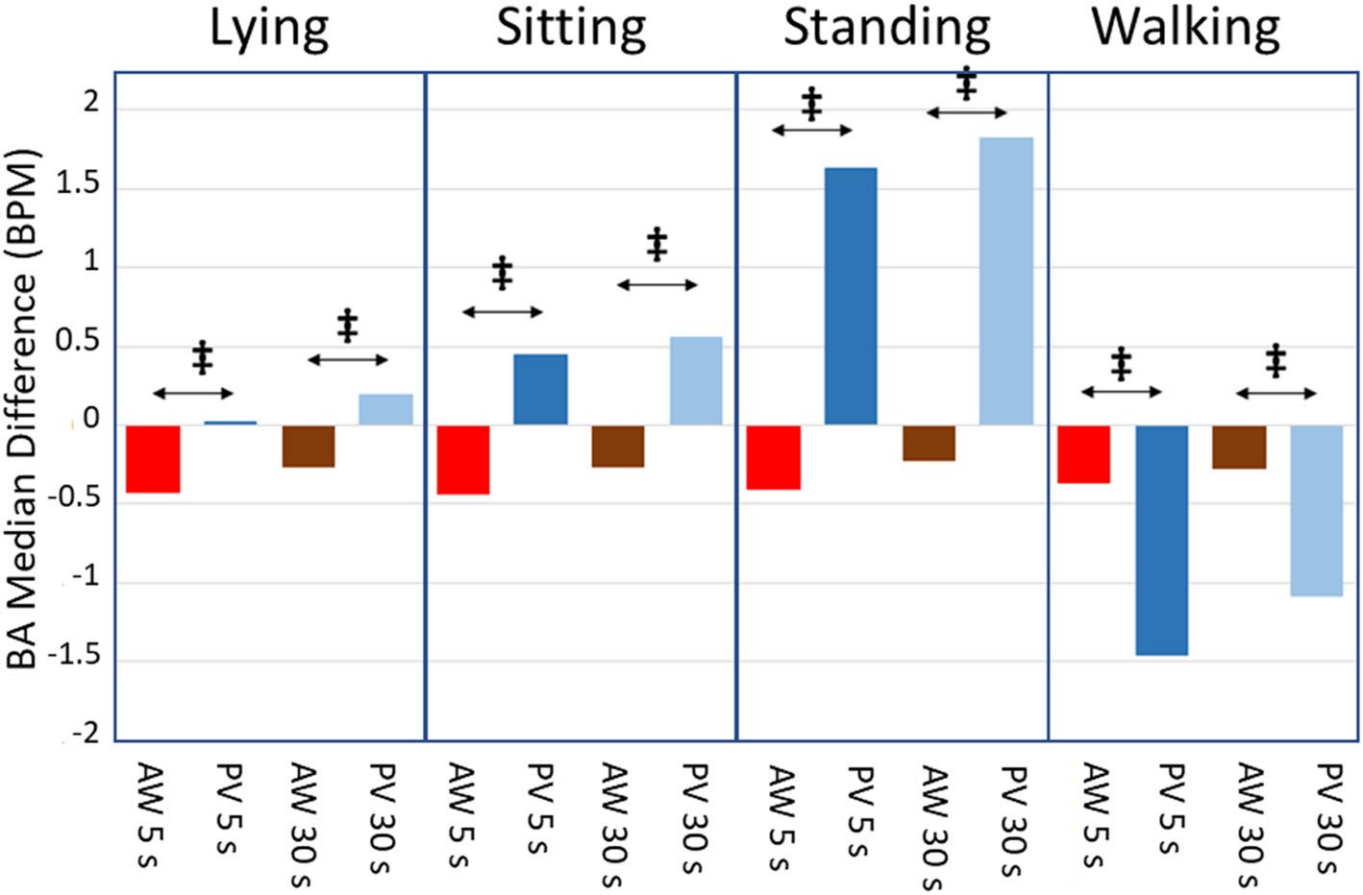
Median differences of the Bland–Altman for the AW and PV devices for 5 s and 30 s averaging time and for the four activities. Red bars are for AW device and 5 s averaging time, dark blue are for PV device and 5 s, brown are for AW device and 30 s and light blue are for PV device and 30 s. Wilcoxon Rank Sum Test results are also shown comparing the median values of differences for both devices. Significant differences: ‡ p < .001.

Figure 4 shows the median differences of the BA when comparing the AW or the PV against the GS for the different activities (lying, sitting, standing and walking) and two averaging times (5 s and 30 s). The figure also shows the statistical significance of the differences in median when comparing the biases of AW and PV against the GS for the different averaging times and activities. All the comparisons show significant differences (p < 0.001, ‡). Note that except while walking, the median differences for AW and PV have opposite sign and that the median difference is always negative for AW. This is a seemingly surprising result considering that results in Fig. 2 predict a statistically significant positive mean difference when averaging for around 5 min. Nevertheless, this sign difference may be attributed to the asymmetrical distribution of averaged HR differences.

**Figure 5:**
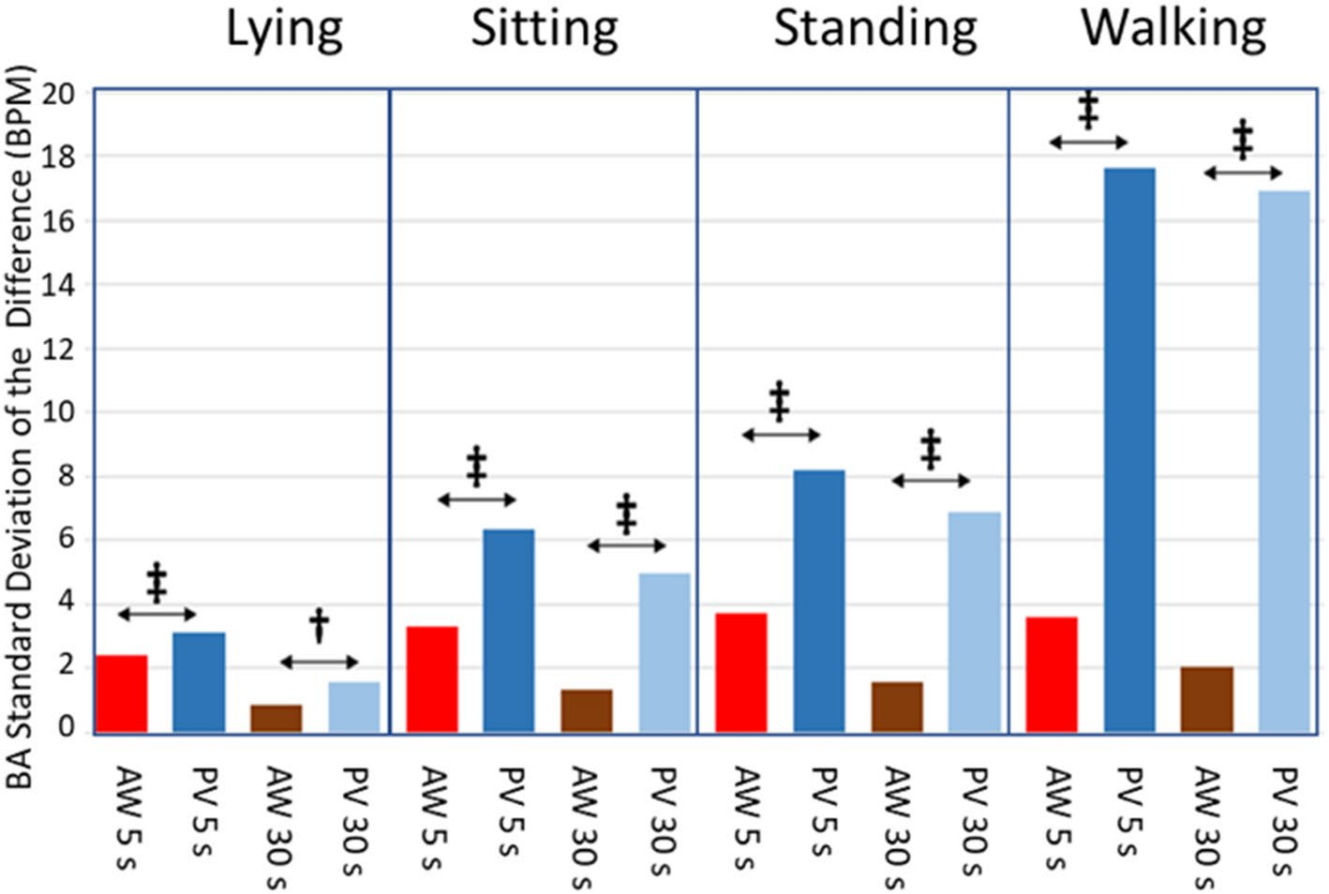
Standard deviation of the differences in the Bland–Altman for the AW and PV devices for 5 s and 30 s averaging time and for the four activities. Red bars are for AW device and 5 s averaging time, dark blue are for PV device and 5 s, brown are for AW device and 30 s and light blue are for PV device and 30 s. Ansari-Bradley Test results are also shown comparing the spread of the differences for both devices. Significant differences: ‡ p < .001; † p < .05.

Figure 5 shows the standard deviation of the differences of the BA to assess the spreading of the differences as well as the statistical significance of the difference between spreads of the BA when comparing the AW and PV against the GS. All the comparisons show significant differences (p < 0.001, ‡) except when comparing AW with PV while lying and when averaging for 30 s (p < 0.05, †). At Appendix B2two tables comparing the bias and spread for the same measuring device during different activities and during different averaging times are provided, also using the Wilcoxon Rank Sum Test and the Ansari-Bradley Test.

Figures 4 and 5 show that when walking while wearing the PV, the median difference change of sign and the standard deviation of the differences increases disproportionately for both averaging times (5 s and 30 s). On the other hand, Fig. 3 shows that when walking, the decrease of the LoA for PV with the averaging time is negligible. That might mostly influenced by the outlier measurements for one of the subjects. Results for walking by removing this subject are shown in Appendix B3.

## Discussion

The first aim of this study was to determine the validity of HR measured by two of the most popular wearables in the market: the Apple Watch and the Polar Vantage, under different levels of activity, compared to a gold standard. Sections "HR agreement by averaging during the whole activity to "Agreement assessed by BA" discuss the Results found regarding this aim. A second goal of the study was to propose a new methodology for testing the validity of HR measurements, which is discussed in Section "Methodology for HR validation".

### HR agreement by averaging during the whole activity

For each activity, mean HR values were obtained and compared between devices. As presented in Fig. 2 and Table 3, mean HR differences between wearables and the GS increased as levels of physical activity rose. Such increase was more evident for the PV than for the AW. That is, the AW values correlated to the GS in all situations (always higher than 0.998), while the correlation of PV and GS decreased as the level of physical activity increased. The Pearson correlation coefficient are in accordance with previous studies^7,27–29^. Other studies comparing watches to ECG found that the accuracy of real-time HR monitoring also reduced as exercise intensity increased^30,31^. For all the wearables and every activity, the correlations were statistically significant.

For reference, lying down was the position in which there were fewer differences between the GS and the other two devices. Nevertheless, while lying down, the biases of mean HR couldn’t be assumed to be zero as pointed by the Bonferroni contrast test for repeated measures, because both the PV and AW provided mean HR significantly lower than the GS. These results are in agreement with^5^ who analyzed people while sleeping, and with^6^ who measured intensive care patients. Both studies reported that wearables tended to slightly underestimate HR at rest. Nevertheless, the differences in mean HR values change from subject to subject in agreement with^26^ and also change when the activity level changes, as can be seen by the different widths of the confidence intervals. Overall, the interindividual differences have more dispersion as the level of activity increases. In this sense, as Table 2 shows, the standard deviations (SD) of the mean values increased as the level of physical activity also increases, being the highest SD (19.42) for PV device in the situation of walking. This is in line with the results of previous studies^7,12^. This rise in dispersion can explain why the Bonferroni contrast test does not provide significant differences for most activities but while lying.

### Agreement assessed by BA

"HR agreement by averaging during the whole activity" Sect. discusses the accuracy of the devices when providing values of HR averaged along the whole duration of each activity (around 5 min) but doesn’t explain how the devices perform for different averaging time intervals, nor their ability to adapt to HR changes throughout the recordings. Figures B1 and B2 in the Appendix B1 shows the BA for averaged HR between pairs of devices, recorded while the volunteers were lying and walking, respectively. Both figures show that the higher the averaging time (*t*_*s*_), the lower the spread of dots is. This means that the agreement between devices increases for larger averaging times. That is reasonable because the longer the averaging the lower the impact of the heart rate variability and noise on the result. The results are summarized in Fig. 3.

Overall, and as expected from results in Fig. 2, the differences between the devices and the GS, especially for PV, are much bigger while walking than when lying, showing a greater sensitivity to the movement in the detection of the HR in the PV versus the AW. This is illustrated by the evolution of the LoA with the averaging time. Figure 3 shows that AW had generally tighter LoA than the PV.

For the PV, the narrowing of the LoA with the averaging time was not so noticeable, especially while walking, meaning that averaging PV’s HR values does not improve the agreement as much as in the case of the AW. This could be attributed to the tendency of the PV to provide unusually higher HR than the GS when there is a certain degree of activity and a limited capability to track changes of the HR as a response to changing physiological states. In fact, it is worth to note that for some measurements the PV was unable to correctly track HR. For example, in Figures B1c and B2d in the Appendix B1 there was an accumulation of dots around a mean value of 125 bpm and mean differences of -80 bpm that corresponded to the results of one of the measured subjects. For this subject, the PV provided readings around 80 bpm higher than the true HR. These readings can be treated as outliers and the origin may be attributable to a modulation of the received light in the photoplethysmograph at twice the stepping cadence associated to the arm’s movement as identified in previous works^15^.

Figure B3 in Appendix B3 replicated the results of Figures B2 and 3 but removing the subject that originated the outliers. The results clearly show that the removal of the outliers mostly affected to the lower bound of the LoA. Nevertheless, the interval defined by the LoA in the PV is still wider than in the AW as seen by Figure B3c. This means that the agreement for AW is better than for PV while walking, regardless the averaging time.

Devices were also compared during different activities and at two averaging times (5 s and 30 s) of HR data obtained. Figures 4 and 5 showed the median differences and the standard deviation of the differences of the BA, respectively, when comparing the AW or the PV. Overall, the spreading of the differences was higher in PV than in the AW, as expected from Fig. 2, confirming that the agreement is better for the AW than for the PV. The dispersion of differences increases with the increase in physical activity, in line with the results of previous studies^7,12^, as mentioned earlier. What was interesting here was that the dispersion reduced when increasing the averaging time. The change of the standard deviation of the differences (or the LoA in Fig. 3) as the averaging time changes is a confounding factor when interpreting the agreement of HR measuring devices. Most studies provide the agreement results when averaging HR during a long and single time (typically 5 min). The current methodology proposed in this study precisely avoids this problem by showing the agreement when averaging at arbitrary intervals: depending on how fast the HR must be updated for a certain experiment, the displayed results in Fig. 3 are useful to estimate the LoA of the measurement.

### Methodology for HR validation

In this study, a methodology to analyze HR data from wearable devices is proposed. This methodology aims to avoid the interpolation of the HR data over longer average times, and instead advocates for a comparison over different time scales to track variations of HR along recording. Interestingly, the proposed synchronization procedure does not require any interpolation of time series, in contrast to recently proposed methods such as in^32^, which promote resampling of time series for delay estimation. In Appendix A4, a study is presented assessing the effects of interpolation using the proposed methodology versus the different time series at 25 Hz as in^32^). The study shows differences in LoA lower than 2 bpm, suggesting it can be of importance only when the compared systems show good agreement.

Moreover, note that the results in this section have considered the pooling of data for every subject. Because differences of averaged HR are also affected by HRV, it is presumable to think that LoA will depend on the HRV of the measured subject. This is especially true for short averaging times. Although Fig. 3 shows the LoA for averaging times up to 60 s, the averaging for longer intervals is straightforward and likely will asymptotically reduce the LoA to values independent of the subject’s HRV. Nevertheless, these values cannot be experimentally obtained due to the finite length of the experiment and the physiological non-stationarity of HR^33^.

### Limitations of the study

Arguably, the main limitation of this study is the small sample size. However, the methodological rigor with which it has been carried out has made it possible to obtain results with a large statistical power. Another limitation is that the wearables being compared were always placed on the same arm. This is a variable that could have affected the results, in that the device on the right arm could register differently from the device on the left. It would be interesting, in future studies, to randomize the positioning of the wearables.

Another aspect to take into account is that, in general, it is considered that a minimum sampling rate is necessary for clinically accurate measurements—30 Hz for HR and 200 Hz for HRV measurements^26^. Nonetheless, these numbers are not very clear, since, for example, a study in patients with cardiovascular disease conclude that the Apple Watch measures HR with clinically acceptable accuracy during exercise, while also stating that it is too early to recommend this device for cardiac rehabilitation^34^. For HR, a main issue comes from the fact that there is no standard measurement. That is: HR is measured by counting beats in a given time window, but the actual way of doing so can differ for each system or software. Given that, we propose, for comparison, to measure the agreement in temporary windows of the same size for all systems, by averaging the HR samples we have for each system within each window. Since there is no a standard of how long the average time should be, we propose to do the analysis for several times (as opposed to most analyzes that use all the observation time).

As a final remark, and despite being effective in accessing HR and HRV, the applications of PPG monitoring are limited by multiple confounders such as sensor pressure against the skin, skin tone, light intensities, and user movement leading to artefactual measurements^14,26,30^. This will have an impact on the feasibility and reliability of mobile phone–based PPG within clinical practice, and should be further explored. In future research it would be interesting to extend the study sample to a wider range of ages and races, as well as take measurements under more demanding physical activities such as running.

## Conclusions

This work analyzed the agreement in HR measurements taken by an Apple Watch (AW) and a Polar Vantage (PV), in comparison to a gold-standard electrocardiogram (ECG), at different activity levels. Results for mean HR values, and at different averaging times, clearly show that the agreement is higher for the AW than for the PV for every activity. Moreover, the best agreement corresponds to the lying position while the worst agreement is found while walking. We conclude that photoplethysmography-based wearable devices are suitable for monitoring HR averages at regular intervals, especially at rest, but their feasibility is debatable for a continuous analysis of HR for research or clinical purposes, especially when involving some level of physical activity. Additionally, this paper proposes a new methodology to synchronize and measure the agreement, against a gold standard, of two or more devices measuring HR at different, and not necessarily uniformly, spaced intervals. This methodology does not require the use of any interpolation or resampling of the data, hence avoiding the need to the unnecessary creation of artificial data and always working using the sampled data provided by the devices. The proposed method also provides an easy way to explore the agreement of the devices at different time scales, allowing to translate the results of the analysis as a function of how much time is devoted to estimate HR. Although the analysis of agreement is based on mean HR differences reported by the devices during a certain time, the extension to other analysis based on statistics such as differences in median, mode, percentiles or extreme values, is straightforward.

## Supplementary Information

Supplementary Information 1.

Supplementary Information 2.

## Data Availability

The datasets generated and/or analysed during the current study are available in the OSF repository, https://osf.io/9x7zs/?view_only=c7536f159f9c48a1ae605319eadaae4a.
