# Supplementary material for: Agreement between two photoplethysmography-based wearable devices for monitoring heart rate during different physical activity situations: a new analysis methodology
Source: Sci Rep. 2022 Sep 14;12:15448. doi: 10.1038/s41598-022-18356-9 (PMC9474518; doi:10.1038/s41598-022-18356-9)
Supplement: Supplementary file 1 — Supplementary Information 1. [file 41598_2022_18356_MOESM1_ESM.docx]

Appendix A: Supplementary information on Signal Processing Stages

A1. QRS detection and Outlier correction

The first step in the QRS detection is to obtain a rough estimate of every QRS wave in the ECG measured by the GS system. Briefly, a first rough fiducial point was obtained using the Pan-Tompkins QRS detector [17] but was further refined by maximizing the correlation between any detected QRS complex and the first detected QRS complex using templates of 200 ms duration centered (100 ms and 100 ms after) on the rough fiducial point.

Because changing posture can have a great impact on the QRS detection, in order to avoid excessive misdetections, we performed separate QRS detections for each one of the measuring situations (lying, sitting, standing and walking) by splitting the ECG using the beginning and ending activity time annotations made by the researchers.

Outliers were corrected similarly as explained in [18]. Briefly, a raw RR time series was obtained by measuring the time intervals between consecutive QRS complexes occurrences. If the change from one RR interval to the next exceeds the interquartile range of the diﬀerentiated RR time series ten-fold then it is decided that an artifact is present*.* The artifact is further classified as false positive (at least two consecutive RR intervals with a value significantly lower than the median of the previous 10 RR intervals), false negative (at least one consecutive RR interval with a value significantly higher than the median of the previous 10 RR intervals) or ectopic beat (two consecutive RR intervals, one with a significantly lower value and the other with a significantly higher value than the median of the previous 10 RR intervals). For a false positive, as many consecutive QRS locations were dismissed as needed to obtain a corrected RR interval with a value near the mean of the previous ten RR intervals. For a false negative, as many artificial QRS locations were introduced uniformly spaced as required to have RR intervals with equal values near to the mean of the previous ten RR intervals. The correction of ectopic beats displaced the QRS location that influences the involved two RR intervals in order to have two identical and consecutive RR intervals.

Hence, for each volunteer and activity, an RR_GS_ was obtained and its timestamp time series was recursively defined as:

$t_{GS}\left( 1 \right)=t_{1} t_{GS}\left( i \right)=t_{GS}\left( i-1 \right)+{RR}_{GS}(i)$ (A1)

where t_1_ corresponds to the time elapsed from the beginning of the recording to the start of the activity and *i* has values ranging from 1 to *N*, the total number of RR time intervals. Once detection of the QRS complexes was completed for each activity, the time series (both RR_GS_ and t_GS_) were concatenated to provide a unique time series for the whole GS recording.

A2. Synchronization

Previously to synchronizing the time series, HR_AW_ and HR_PV_ were converted to averaged RR time series (aRR_AW_ and aRR_PV_) by using the following transformations:

${aRR}_{AW}\left( j \right)=\frac{60000 ms/minute}{{HR}_{AW}(j)}$ ${aRR}_{PV}\left( k \right)=\frac{60000 ms/minute}{{HR}_{AW}(k)}$ (A2)

To synchronize the aRR_AW_ with the corresponding RR_GS_ time series is simply to add to t_AW_ a proper constant representing the delay with respect to t_GS._ Because the sampling interval for the time series is different (for the PV is constant and at 1 second per point estimate, AW is irregular between 1 to 9 s, and GS is unevenly sampled at every time a heartbeat occurs), an error metric was defined by measuring the difference between each RR_GS_ sample with respect to the nearest in time aRR_AW_ sample as indicated by a delayed version of the timestamp t_AW_ time series. The synchronization procedure estimates the necessary delay that minimizes the differences between RR_GS_ and aRR_AW_ time series.

Let’s consider the delayed timestamp time series defined as:

${td}_{AW}\left( j \right)=t_{AW}\left( j \right)+\Delta t$ (A3)

where $\Delta t$ is the delay. The error metric is computed for each RR_GS_ sample as:

$e_{\Delta t}=\sum_{i=1}^{N} \left( {RR}_{GS}\left( i \right)-{aRR}_{AW}(j_{nearest}) \right)^{2}$ (A4)

The difference for each ${RR}_{GS}$ sample is obtained by finding the closest sample in time (*j_nearest_*) of the ${td}_{AW}$ time series to t_GS_(i). The $e_{\Delta t}$ statistic adds these differences for all the available ${RR}_{GS}$ samples.

The synchronization procedure tests some candidate values of $\Delta t$ and chooses the one that minimizes (A4). Accordingly, the synchronized AW timestamp time series (t_SAW_) is obtained by minimizing $e_{\Delta t}$ over a sufficient wide range of $\Delta t$. Accordingly to this procedure, an optimal delay is found as:

$t_{SAW}\left( j \right)=\arg\min_{\Delta t} e_{\Delta t}$ (A5)

And the t_SAW_ time series is defined as $t_{SAW}\left( j \right)=t_{AW}\left( j \right)+t_{SAW}\left( j \right)$ (A6)

Because delays among starting measurement systems and processing times can be higher than 10 s, in this work we have tested for each subject delays from -100 s to 100 s with a resolution of 1 s. We have empirically observed that increasing the delay resolution to 10 ms did not change significantly the minimum value of $e_{\Delta t}$, hence we opted for the resolution of 1 s to reduce the processing time. For the PV, another synchronized PV timestamp time series (t_SPV_) has been also obtained following the same procedure as in the case of the AW. Figure A1 shows an example of how the three times series are aligned after synchronization.


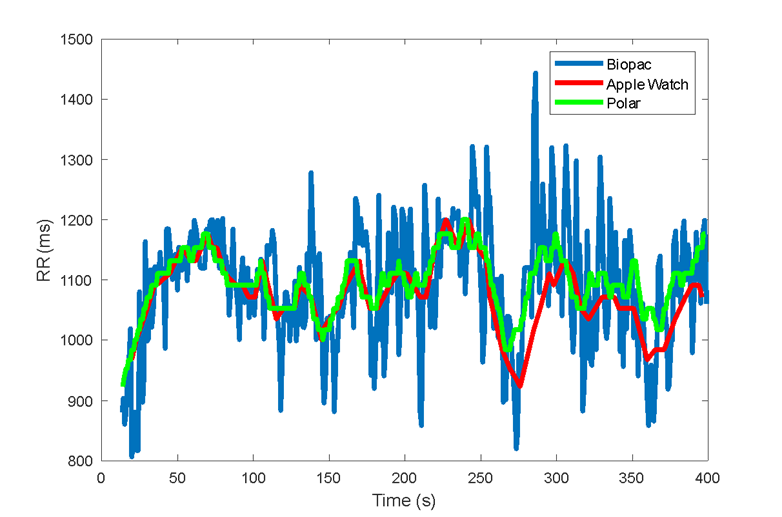


**Figure A1.** Example of synchronization of the AW and PV derived RR series with the RR series obtained from the ECG of subject 1 while lying still.

A3. An example of averaging

Figure A2 shows an example on how the averaging procedure is done using an averaging time of 5 s. The figure markers show the pairs (t_sAW_(j),aRR_AW_(j)) as green circles, (t_sPV_(k),aRR_PV_(k)) as red crosses and (t_GS_(i),RR_GS_(i)) as blue asterisks for a subject s while lying. For the interval starting at t= 252 s and finishing at t= 257 s, $\bar{{aRR}_{AW}^{s,lying,5 s}\left( m \right)}$ will be computed by averaging the two samples of aRR_AW_ inside the interval, $\bar{{aRR}_{PV}^{s,lying,5s}\left( m \right)}$ will be estimated by averaging 5 samples of aRR_PV_ while $\bar{{aRR}_{GS}^{s,lying,5s}\left( m \right)}$will be obtained by averaging 3 samples of RR_GS._ The next interval for averaging ($m$+1) starts at t= 253 s and ends at t= 258 s. The averaging procedure uses 1 sample of aRR_AW_ , again 5 samples of aRR_PV_ and 4 samples of RR_GS_. The interval starting at t= 254 s and ending at t= 259 s is skipped because there are no available samples for AW. Note that the number of samples averaged at each interval is different for each device and only constant for PV because it is the only device that provides samples at a constant rate. Nevertheless, the averaging is performed for every device corresponding to the available measures inside the same time interval.


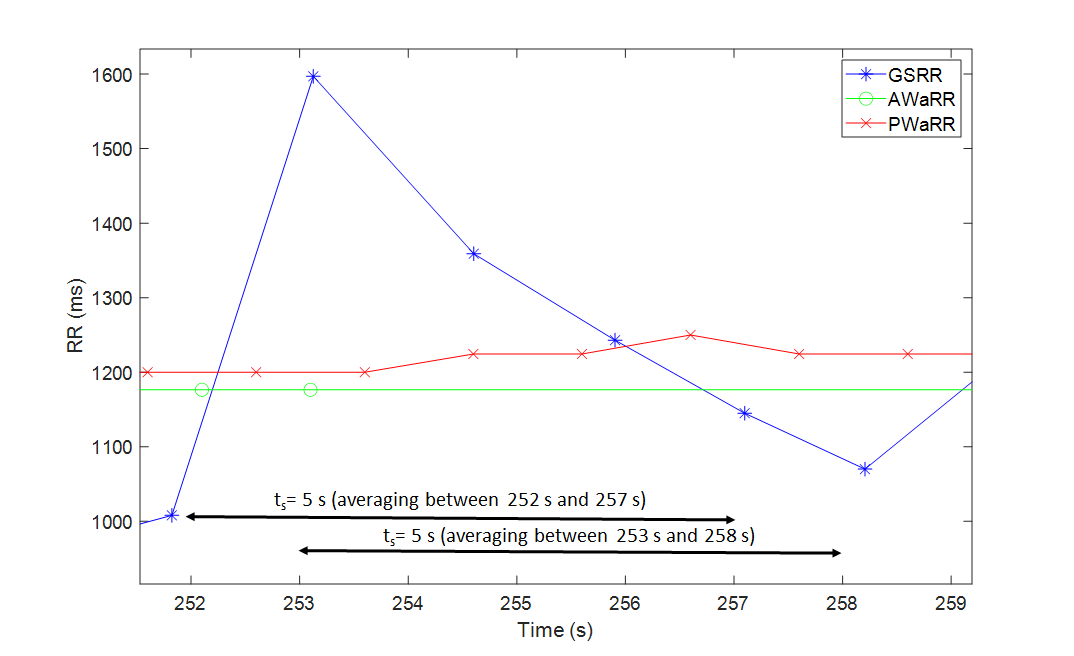


RR_GS_

aRR_AW_

aRR_PV_

**Figure A2.** Example of the averaging procedure for *t_s_* =5 s. See text for a detailed description.

A4. The effect of interpolation in LoA

In most studies where systems with different sampling strategies are compared, the measured time series are resampled (usually using linear interpolation) and after this procedure the differences between measuring systems are obtained. In this study, we have repeated the measurement of LoA but using the resampled versions of the original time series using linear interpolation. The resampling frequency has been 25 Hz as in reference [30]. The difference in 2.5% and 97.5% percentiles with respect to the results without using resampled time series for each averaging time have been computed when comparing the PV or the AW to the gold standard for the different activities. These differences are displayed in Figure A3 for the AW and in Figure A4 for the PV. Results show that the differences are modest (always lower than 2 bpm) and particularly low while walking. Probably, this could be justified by the lower HRV during this activity. Moreover, and according to the sign of the differences, the interpolation procedure seems to provide values of the percentiles lower than without using interpolation, hence the resampling introduces a bias that can be significant for systems showing good agreement.

Sitting

Lying

Walking

Standing

**Figure A3.** Differences in LoA associated to the linear interpolation of the time series when comparing the results of AW versus the Biopac system. The differences have been computed as the LoA obtained without interpolation minus the LoA obtained after resampling the time series at 25 Hz using linear interpolation. The blue trace shows the differences for the 2.5% percentile while the red trace shows the differences for the 97.5% percentile.

Sitting

Lying

Walking

Standing

**Figure A4.** Differences in LoA associated to the linear interpolation of the time series when comparing the results of PV versus the Biopac system. The differences have been computed as the LoA obtained without interpolation minus the LoA obtained after resampling the time series at 25 Hz using linear interpolation. The blue trace shows the differences for the 2.5% percentile while the red trace shows the differences for the 97.5% percentile.
