# Supplementary material for: Agreement between two photoplethysmography-based wearable devices for monitoring heart rate during different physical activity situations: a new analysis methodology
Source: Sci Rep. 2022 Sep 14;12:15448. doi: 10.1038/s41598-022-18356-9 (PMC9474518; doi:10.1038/s41598-022-18356-9)
Supplement: Supplementary file 2 — Supplementary Information 2. [file 41598_2022_18356_MOESM2_ESM.docx]

Appendix B: Supplementary Information for BA results

B1. BA plots for selected activities and averaging times

Figure B1 shows the BA for averaged HR between pairs of devices, recorded while the volunteers were lying. The differences between data from the Biopac (GS) and the Apple Watch (AW) are shown in Figures B1a and B1b for averaging times (*t_s_*) of 5 s and 30 s, respectively. As described by equation (5), each dot in the figure represents the arithmetic mean (x axis) against the difference (y axis) of the averaged heart rate in bpm of each device when averaged using 5 s or 30 s epochs, respectively. The differences between GS and Polar Vantage (PV) are shown in Figures B1c and B1d, with also an averaging time of HR in epochs of 5 s and 30 s, respectively. The black line in each plot denotes the median of the differences while the two red lines correspond to the percentiles 2.5% and 97.5%. We have used percentiles to characterize the limits of agreement because their distributions can be long tailed and asymmetrical.

Figure B2 shows the BA for the walking activity. The differences between data from the Biopac (GS) and the Apple Watch (AW) are shown in Figures B2a and B2b for averaging times (*t_s_*) of 5 s and 30 s. The differences between GS and Polar Vantage (PV) are shown in figures B2c and B2d, with also an averaging time of HR in epochs of 5 s and 30 s, respectively. The differences with respect to the GS, specially the PV, are much bigger while walking than when lying.


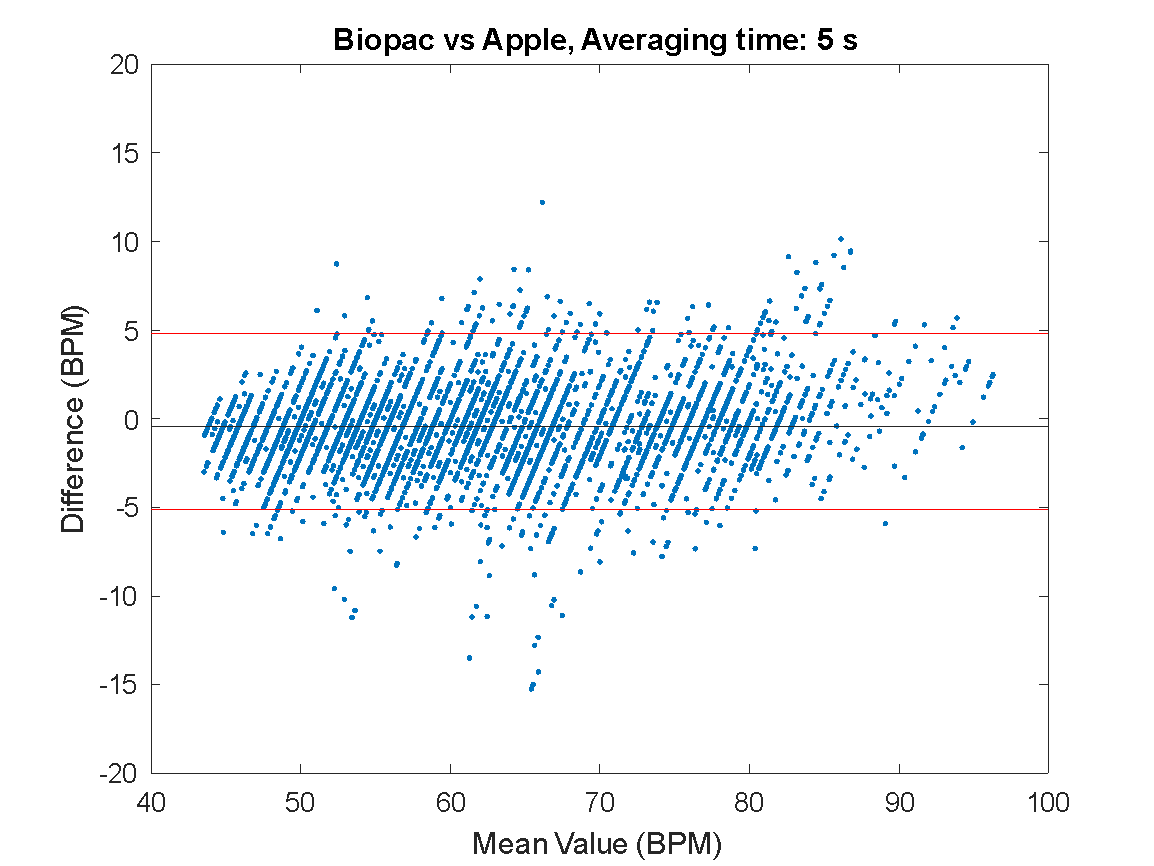

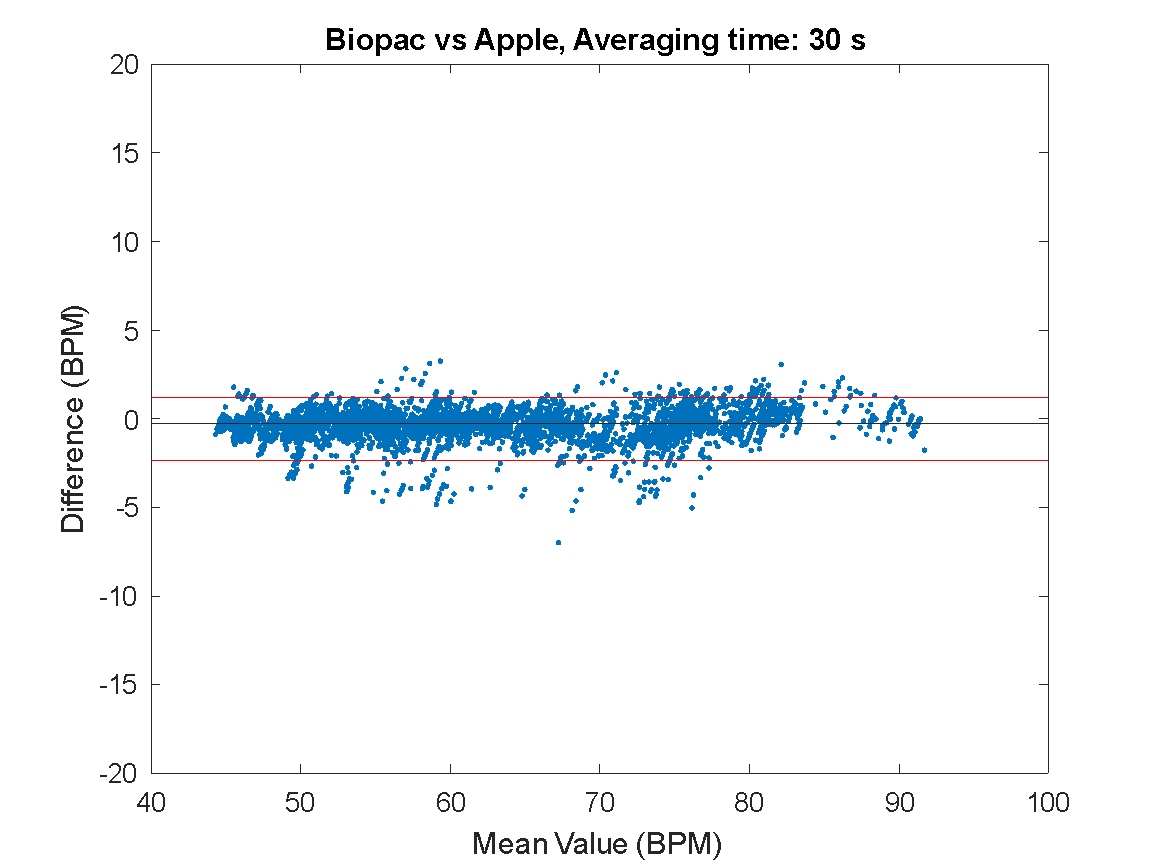

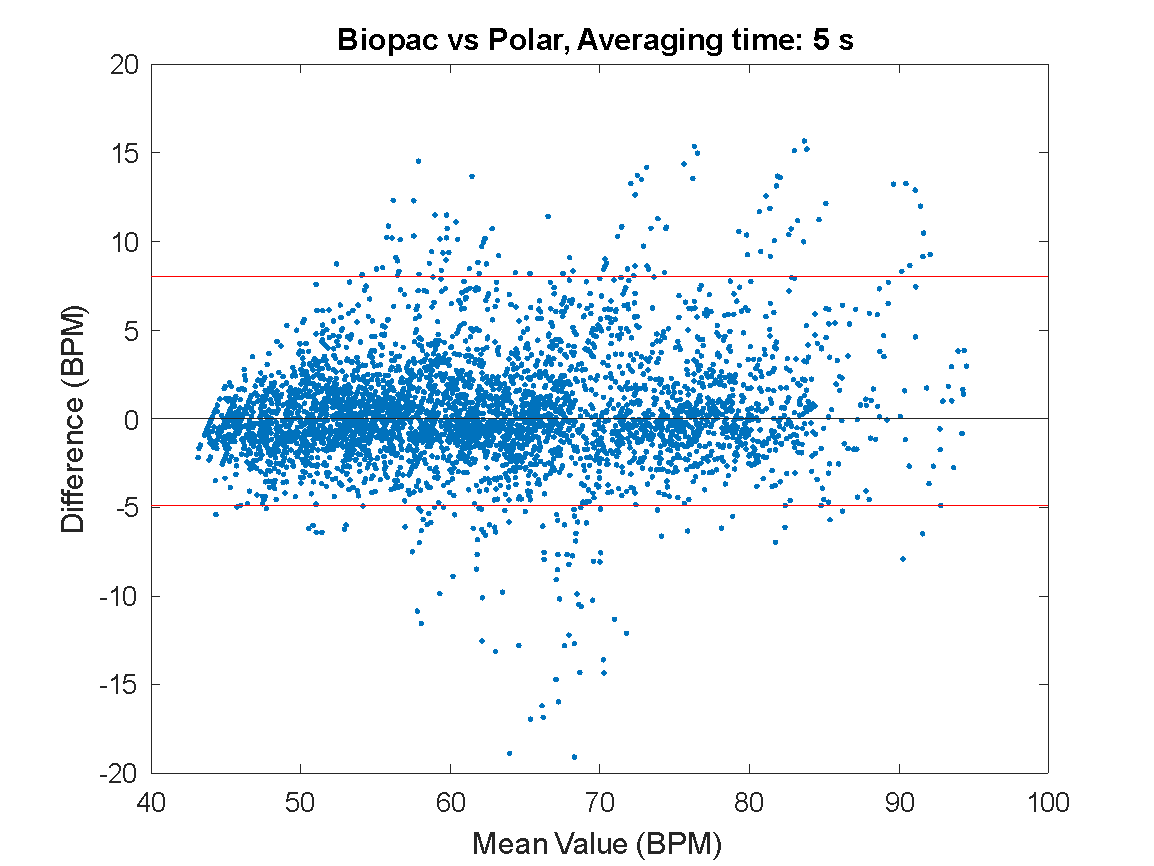

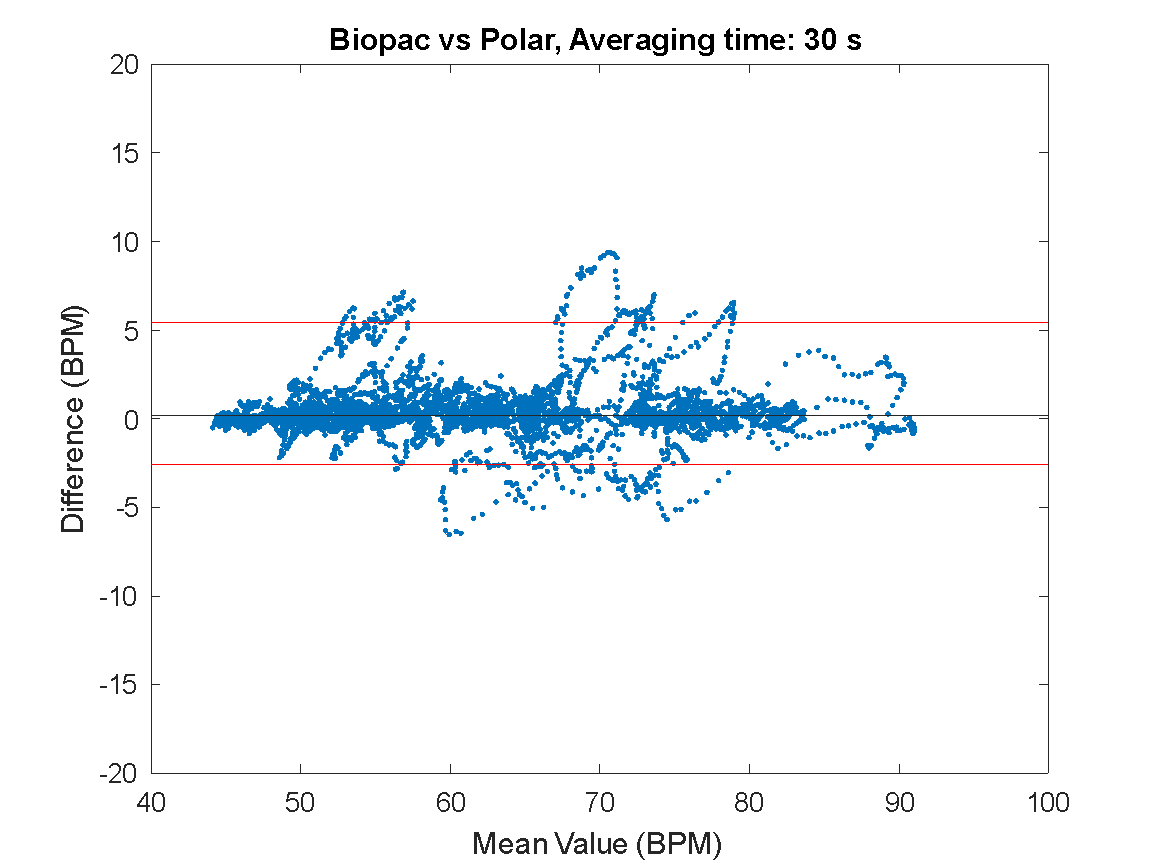


a

b

c

d

**Figure B1.** Bland-Altman plots of averaged HR while lying for 5 s and 30 s averaging times. Figures a and b are for AW vs GS, and Figures c and d are for PV vs GS. The red traces represent the limits of agreement (LoA) computed as the 2.5% and 97.5% percentiles of the differences with respect to the GS while the black trace shows the median of the differences.

**Figure B2.** Bland-Altman plots of HR while walking for 5 s and 30 s averaging times for AW vs GS (a,b) and PV vs GS (c,d). The red traces represent the limits of agreement (LoA) for the 2.5% and 97.5% while the black trace shows the median of the differences


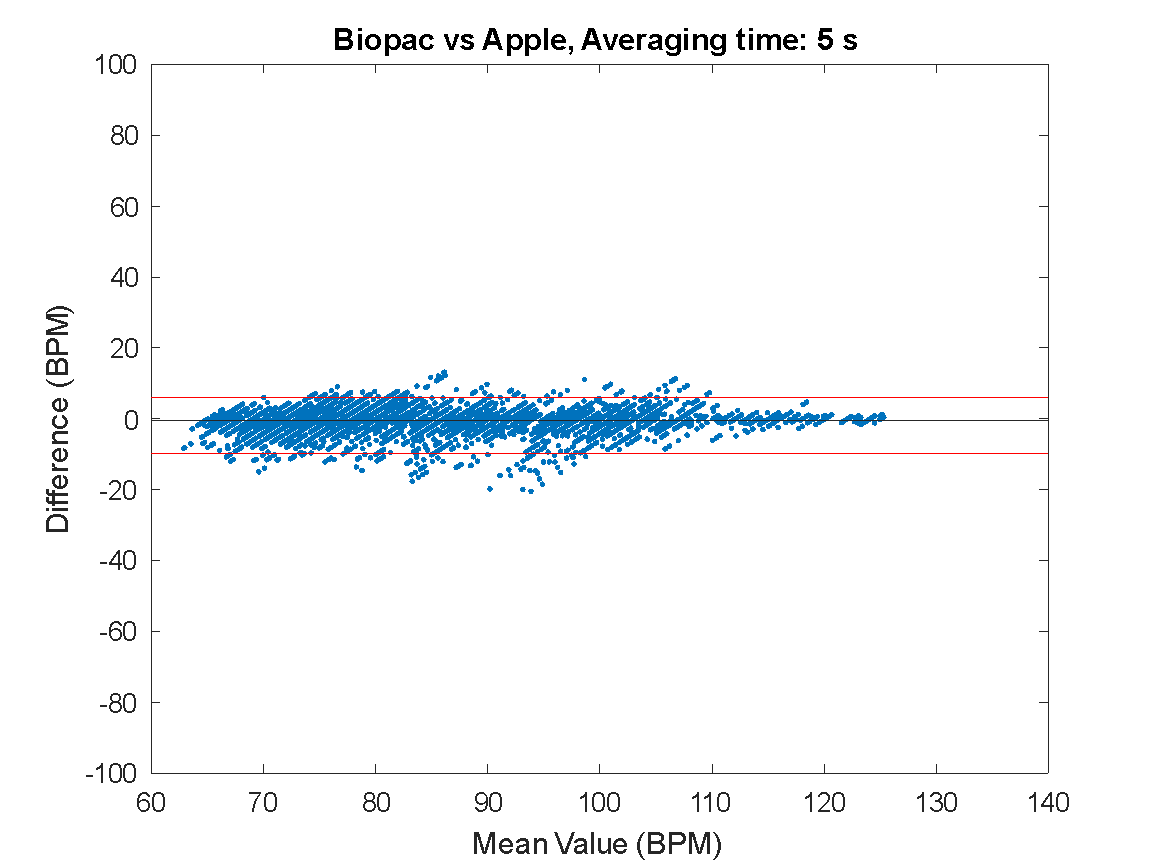

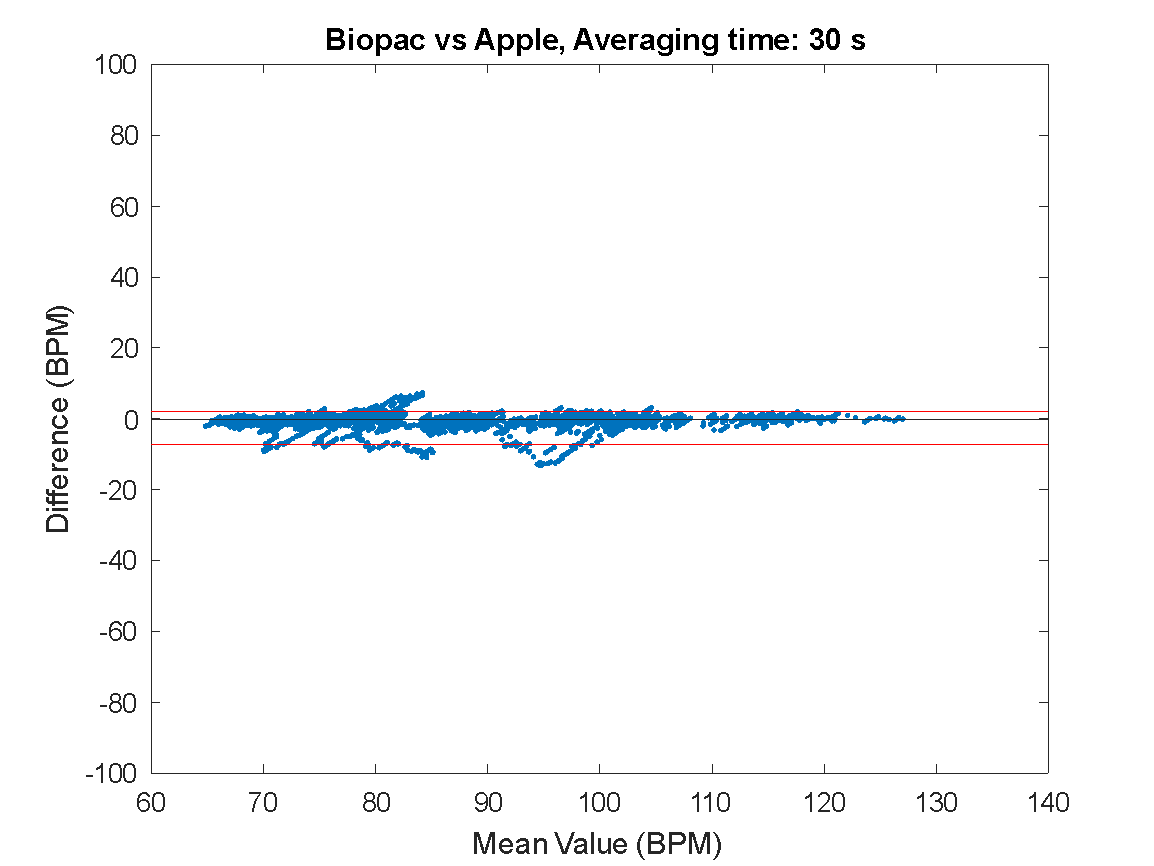

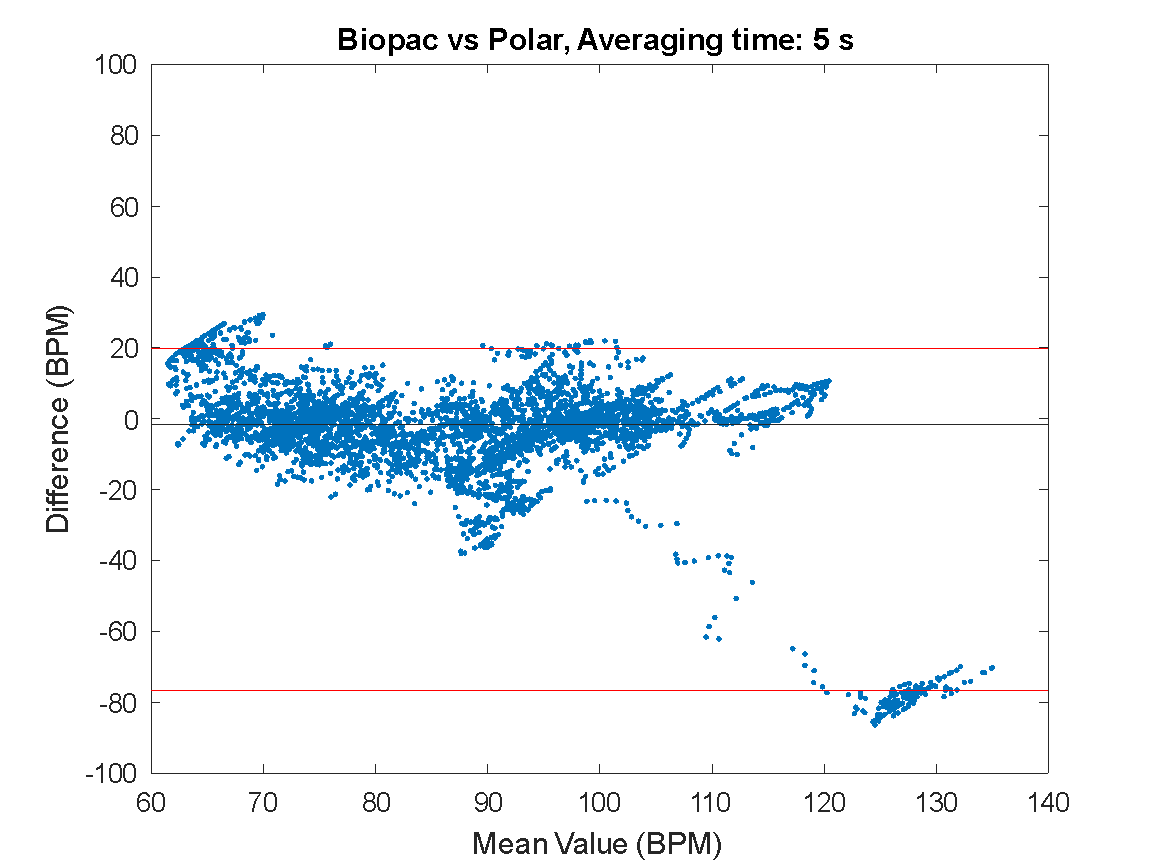

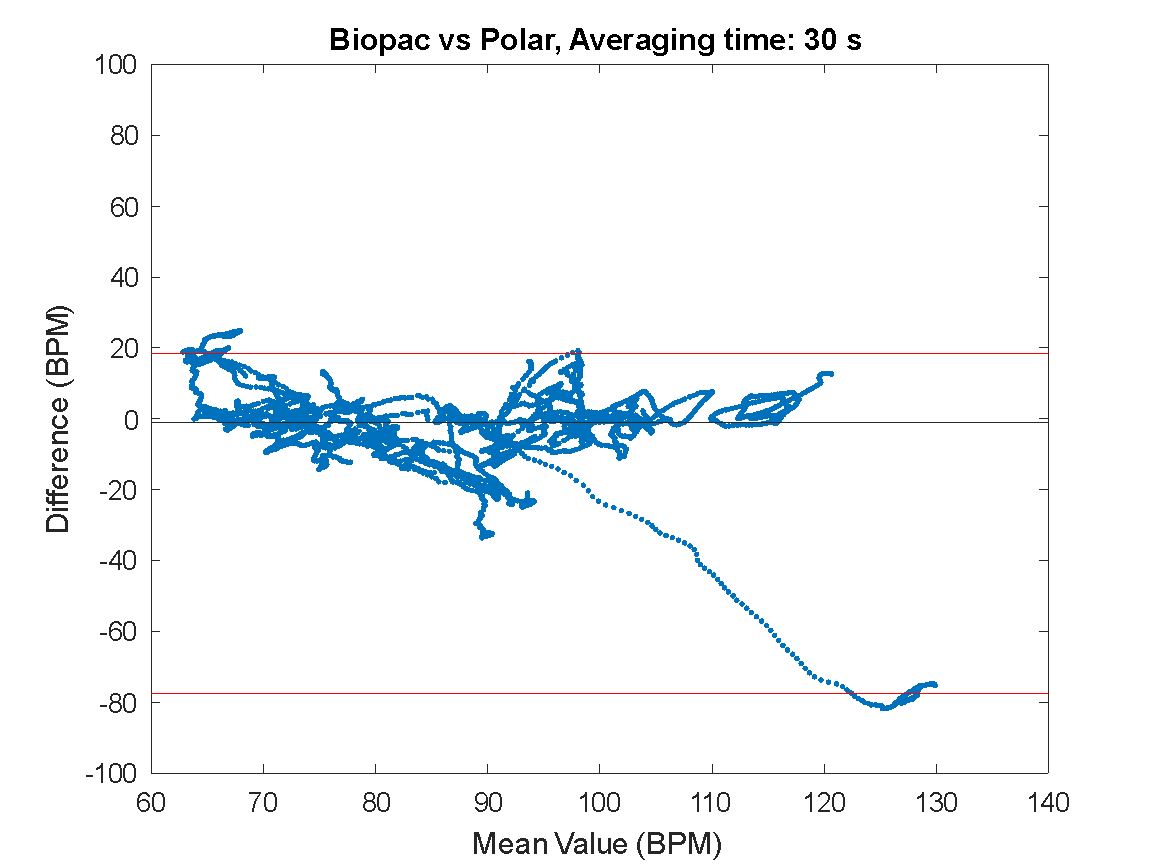


a

b

c

d

B2. Other BA statistical test results

Table 4 provides the statistical significance of differences when comparing the bias (Wilcoxon Rank Sum Test) and spread (Ansari-Bradley Test ) of the BA for the same device during different activities using the same averaging time while table 5 provide the same results for the same device and activity using the 5 s or the 30 s averaging time.

**Table B1.** Statistical significance of differences in bias and spread separated by device at the same averaging time for different activities.

|  | Bias AW | | Spread AW | | Bias PV | | Spread PV | |
| --- | --- | --- | --- | --- | --- | --- | --- | --- |
|  | *t_s_*= 5 s | *t_s_*= 30 s | *t_s_*= 5 s | *t_s_*= 30 s | *t_s_*= 5 s | *t_s_*= 30 s | *t_s_*= 5 s | *t_s_*= 30 s |
| Lying vs Sitting | n.s. | n.s. | ** | ** | ** | ** | ** | ** |
| Lying vs Standing | n.s. | ** | ** | ** | ** | ** | ** | ** |
| Lying vs Walking | n.s. | n.s. | ** | ** | ** | ** | ** | ** |
| Sitting vs Standing | n.s. | * | n.s. | ** | ** | ** | ** | ** |
| Sitting vs Walking | n.s. | n.s. | n.s. | ** | ** | ** | ** | ** |
| Standing vs Walking | n.s. | ** | ** | ** | ** | ** | ** | ** |

Significant differences: n.s. p>0.01, *p<.01; **p<.001**

**Table B2.** Statistical significance of differences in bias and spread separated by device and activity when comparing averaging times *t_s_*= 5 s vs *t_s_*= 30 s.

|  | Bias AW | Spread AW | Bias PV | Spread PV |
| --- | --- | --- | --- | --- |
| Lying | ** | ** | ** | ** |
| Sitting | n.s. | ** | ** | ** |
| Standing | * | ** | ** | ** |
| Walking | n.s. | ** | n.s. | ** |

Significant differences: n.s. p>0.01, *p<.01; **p<.001**

B3. Results for walking after removing one subject from the pooling

Figure B3 replicates the results of Figure 6c, 6d and the evolution of LoA when walking for PV of Figure 3 by removing the subject that provided wrong measurements. After removal of the outlier the median of the differences is -1.14 bpm for *t_s_* = 5 s and -0.81 bpm for *t_s_* = 30 s while the standard deviation of the differences is 9.94 bpm for *t_s_* = 5 s and 8.91 bpm for *t_s_* = 30 s. Median and standard deviation of the differences are significantly (p<0.001) different when compared to results for the AW.

 a b

c

**Figure B3.** Bald-Altman plots and LoA while walking while removing the subject with outlier measurements. Figure B3a shows the Bald-Altman plot for an averaging time of 5 s while Figure 10b corresponds to an averaging time of 30 s. Figure b3c shows the change of LoA (percentiles 2.5% and 97.5%) with the averaging time for the PV in red and the AW in black.
